# Unlocking RAS: Finding the Right Combination Is the Key

**DOI:** 10.1002/ueg2.70138

**Published:** 2025-10-21

**Authors:** Bjoern Papke, Channing J. Der

**Affiliations:** ^1^ Institute of Pathology Charité – Universitätsmedizin Berlin Berlin Germany; ^2^ German Cancer Consortium (DKTK) Partner Site Berlin German Cancer Research Center (DKFZ) Heidelberg Germany; ^3^ Lineberger Comprehensive Cancer Center University of North Carolina at Chapel Hill Chapel Hill North Carolina USA; ^4^ Department of Pharmacology University of North Carolina at Chapel Hill Chapel Hill North Carolina USA

**Keywords:** EGFR, ERBB2/HER2, KRAS oncogene, receptor tyrosine kinase (RTK), rectal adenocarcinoma (RC)

The belief that RAS was “undruggable,” once etched in stone, was shattered with the first clinical approval of KRAS^G12C^ inhibitors in 2021 and 2022. Since then, numerous RAS inhibitors that target different oncogenic mutations, including pan‐RAS approaches, have entered clinical trials [1]. However, colorectal cancer (CRC) poses a significant therapeutic challenge for RAS inhibition due to robust feedback loops and compensatory receptor tyrosine kinase (RTK) activation, similar to resistance mechanisms previously observed in the early years of BRAF inhibitor clinical development [2, 3]. Consequently, combination therapy with EGFR inhibitors is essential to counteract these adaptive responses, an approach recently validated for KRAS^G12C^ inhibitors [4, 5]. Nevertheless, responses to the combination of KRAS^G12C^ inhibitors with EGFR antibodies remain incomplete, and resistance typically develops with a progression free survival of approximately 7 months [6].

In this issue of the United European Journal of Gastroenterology, Buchloh et al. investigate combination therapies for RAS inhibitor treatment specifically in rectal cancer (RC), which comprises one‐third of all colorectal cancer [7]. Notably, approximately half of RC harbor a RAS mutation, with 45% carrying KRAS mutations and 5% NRAS mutations (cBioPortal [MSK RC dataset]). The authors first to set out to investigate the clinical impact of RAS mutations on the patient's outcome after neoadjuvant therapy. In their Goettingen rectal cancer cohort (390 patients), they found that the disease‐free survival was significantly reduced in rectal cancer patients carrying KRAS^G12C^ and KRAS^G12V^ mutations compared to KRAS wild‐type patients.

Given the emergence of clinically approved KRAS^G12C^ inhibitors and the reduced survival associated with KRAS^G12C/V^ mutant RC, the authors turned to rectal cancer cell line models to investigate mechanisms of resistance and to identify effective combination strategies. In both model RC cell lines, SW1463 and SW837, they observed rapid adaptive resistance to RAS inhibition, consistent with previous findings in colon cancer. To systematically address this resistance, the authors performed a drug screen of 125 compounds in both cell lines. This drug screen identified eight candidate inhibitors that were effective in both models, targeting IGF‐1R, ERBB2/EGFR, MEK, PRMT1, SHP2, farnesyltransferase, SOS, and MDM2. Notably, the majority of these targeted either RTKs (ERBB2, EGFR, and IGF‐1R) or key signal components connecting RTKs with RAS (SOS1 and SHP2) and RAS effector signaling (MEK), highlighting recurrent vulnerabilities in RAS‐mutant contexts and underscoring the pivotal role of RTK‐RAS pathway reactivation in RC (Figure 1).

**FIGURE 1:**
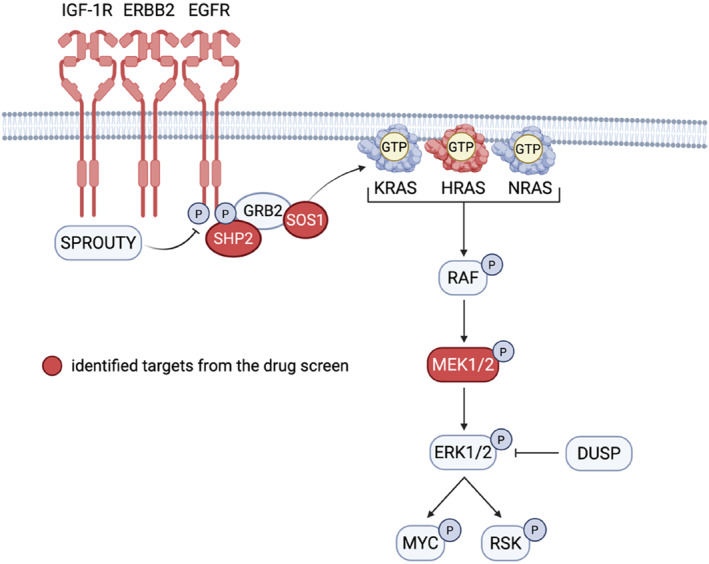
Drug screen reveals recurrent vulnerabilities in RTK‐RAS/MAPK signaling. Schematic overview of the RTK–RAS/MAPK signaling pathway, indicating key nodes identified in the drug screen (red). Figure created with BioRender.com.

Subsequent combination studies using the approved KRAS^G12C^ inhibitor sotorasib, the pan‐KRAS inhibitor BI‐2865, and the clinical candidate multi‐RAS(ON) inhibitor daraxonrasib/RMC‐6236 together with the pan‐HER inhibitor neratinib demonstrated robust and consistent synergistical enhancement of proliferation inhibition across various assays and models including organoid cultures, further supporting the robustness of these combination approaches.

In conclusion, this study underscores the critical role of RTKs in RC and highlights that RAS inhibition alone is unlikely to be sufficient. Therefore, more effective combination strategies will be necessary to sensitize tumor cells and to overcome resistance.

Finally, the concept of the Goldilocks principle appears to apply to RTK inhibition as well: too little inhibition leads to rapid resistance, while excessive inhibition, such as that seen clinically with SHP2 and SOS inhibitors as pan‐RTK inhibitors, can result in adverse effects. However, we are only beginning to discover what “just perfect” means in terms of RAS‐RTK inhibition and which RTKs will play major roles.

## Conflicts of Interest

C.J.D. is a consultant/advisory board member for AskY Therapeutics, Cullgen, Deciphera Pharmaceuticals, Mirati Therapeutics, Reactive Biosciences, Revolution Medicines and SHY Therapeutics. C.J.D. has received research funding support from Deciphera Pharmaceuticals, Mirati Therapeutics, Reactive Biosciences, Revolution Medicines, and SpringWorks Therapeutics. The other author declares no competing interests.

## Data Availability

The authors have nothing to report.
